# Implications of attention‐deficit/hyperactivity disorder diagnostic timing on mental health service utilisation in young adult females: A population‐based record linkage cohort study

**DOI:** 10.1002/jcv2.70133

**Published:** 2026-06-03

**Authors:** Estelle Alderson, Nicholas Bowden, Laurie McLay, Ben Beaglehole, Philip J. Schluter

**Affiliations:** ^1^ Faculty of Health University of Canterbury Christchurch New Zealand; ^2^ Department of Paediatrics and Child Health University of Otago Dunedin New Zealand; ^3^ Department of Psychological Medicine University of Otago Christchurch New Zealand; ^4^ School of Clinical Medicine Primary Care Clinical Unit The University of Queensland Brisbane Queensland Australia

**Keywords:** attention‐deficit/hyperactivity disorder, diagnostic timing, epidemiology, mental health service utilisation, young adult females

## Abstract

**Background:**

Sex differences in the frequency and timing of attention‐deficit/hyperactivity disorder (ADHD) diagnosis exist. While there are multiple known individual and wider consequences for young females with delayed or undiagnosed ADHD, little is known about mental health service utilisation (HSU) impacts.

**Methods:**

A national population‐based cohort of females aged 18–24 years between July 1, 2021, to June 30, 2022, in Aotearoa New Zealand was established. ADHD‐index, intellectual disability (ID)‐index and age at index were derived using established algorithms. Four mental and two non‐mental health variables capturing HSU interactions between July 1, 2022, to June 30, 2024 were derived and analyzed for ADHD‐index participants. Unadjusted and adjusted modified Poisson regression models were utilised and reported. All analyses were stratified by ID‐index.

**Results:**

Overall, 212,385 females were included, of whom 5244 (2.5%) had ADHD‐index. Of those ADHD‐indexed, 486 were also ID‐indexed. For females ADHD‐indexed, 1638 (31.2%) were indexed at age < 12 years, 1575 (30.0%) at age 12–17 years, and 2031 (38.7%) at age 18–24 years. While different patterns emerged between females with and without ID, those with later ADHD diagnosis were associated with higher rates of psychotropic pharmaceutical dispensing, psychiatric inpatient stays, and non‐psychiatric hospitalisations compared to those with early diagnosis. For example, in the adjusted analysis comparing females without ID‐index who were ADHD‐indexed at age 12–17 years to their counterparts indexed at age < 12 years, the estimated incident rate ratio of psychiatric inpatient stay was 2.07 (95% CI 1.20–3.58).

**Conclusion:**

Later ADHD diagnosis among a national cohort of young adult females was common. A later diagnosis was associated with increased mental and non‐mental HSU compared to those with earlier diagnosis. Earlier detection of ADHD in females may mitigate their personal burden and reduce subsequent service use.

## Key Points

What's known?

- Attention‐deficit/hyperactivity disorder (ADHD) is a prevalent neurodevelopmental condition among children and adolescents. However, many females experience undiagnosed or delayed ADHD diagnosis which may have individual and societal costs.

**What's new?**

- Scant population data exist that relate diagnosis timing of ADHD in young females to mental health service utilisation (HSU).
- Patterns were different for those with and without intellectual disability. Nonetheless, overall, females with later ADHD diagnosis were associated with higher rates of psychotropic pharmaceutical dispensing, psychiatric inpatient stays, and psychiatric outpatient visits compared to those with early diagnosis.

**What's relevant?**

- While undiagnosed or delayed ADHD diagnosis in females has individual costs, these may also result in higher downstream mental HSU burdens. Efforts aimed at earlier detection and treatment may reduce service use, in addition to mitigating the personal burden.

## INTRODUCTION

Attention‐deficit/hyperactivity disorder (ADHD) is prevalent, affecting approximately 6%–10% of children and adolescents worldwide (Ayano et al., 2023). ADHD prevalence among children and adolescents has increased steadily over past decades, and continued growth is projected (Fan et al., 2025).

Characterised by persistent and pervasive patterns of inattention and/or hyperactivity‐impulsivity (American Psychiatric Association, 2022), ADHD can significantly affect both functioning and development, thereby imposing substantial life‐long challenges (French et al., 2024). ADHD is attributed to complex interactions between genetic, neurobiological, and environmental factors (Hamed et al., 2015), and often co‐occurs with other psychiatric conditions (Centers for Disease Control and Prevention, 2024a). The relationship between ADHD and mental health conditions is complex and multi‐faceted, with characteristics of ADHD both contributing to and exacerbated by adverse mental health outcomes (French et al., 2024). Pharmacological treatments (particularly stimulants and atomoxetine), psychosocial therapies, and their combination have been well‐established as effective interventions for those with ADHD (Pelham & Altszuler, 2020). These can be found in many clinical practice guidelines, including those for the United States of America (USA) (Centers for Disease Control and Prevention, 2024b), United Kingdom (UK) (National Institute for Health and Care Excellence, 2018), and Aotearoa New Zealand (NZ) (Ministry of Health, 2025b).

Within NZ, according to the Ministry of Health's *Clinical Principles Framework for Attention Deficit Hyperactivity Disorder* published in July 2025, ADHD is typically diagnosed in children aged 5–14 years; although in the update published in September 2025, this statement no longer appears (Ministry of Health, 2025b). For diagnosis, it is recommended that characteristics of ADHD are established as pervasive, across time and multiple settings, by age 12 years (Ministry of Health, 2025b). While it is argued that early diagnosis and treatment is effective in minimising downstream negative consequences (Ayano et al., 2023; Fan et al., 2025; Martin et al., 2026; Siddiqui et al., 2024; Tam et al., 2025), variations in professional recognition and assessment contribute to underdiagnosis, misdiagnosis and subsequent treatment delay (Hamed et al., 2015). The increasing ADHD prevalence rates have sparked debate over potential overdiagnosis and overtreatment, particularly in cases involving mild or ambiguous symptoms (Kazda et al., 2021). Concerns also include the medicalisation of normative childhood behaviors, especially among younger children, and the use of pharmacological interventions without first providing adequate behavioral support (Searight & McLaren, 1998). Allocating scarce treatments to mild cases may exacerbate inequities by limiting access for underdiagnosed populations (Abdallah et al., 2025; Kazda et al., 2021).

Significant sex differences in frequency and timing of diagnosis exist (Ayano et al., 2023; Fan et al., 2025). Numerous explanations have been proffered, including: differences in clinical presentation, psychiatric co‐occurrences, diagnostic criteria biases and overshadowing, alongside sociocultural factors (e.g., compensation, masking, and scaffolding) (Almekhlafi & Jain, 2024; Martin, 2024). Females are more likely to report inattentiveness and have co‐occurring depression and anxiety than males, who are more likely to exhibit greater externalised hyperactivity and impulsivity (Almekhlafi & Jain, 2024; Centers for Disease Control and Prevention, 2024a). Without timely support, these internalised symptoms may lead females to experience low self‐esteem, sense of powerlessness, heightened emotional dysregulation, difficulties maintaining relationships, and self‐destructive behaviors (Attoe & Climie, 2023; Young et al., 2020). In addition to the personal burden, diagnostic delays likely carry significant societal costs for females, including excess health service utilisation (HSU) (Martin et al., 2026; Siddiqui et al., 2024; Skoglund et al., 2024).

While research investigating diagnostic timing disparities in females and subsequent HSU has been called for (Almekhlafi & Jain, 2024; Skoglund et al., 2024), population‐based quantitative evidence is scant. Indeed, we are aware of only one study (Martin et al., 2024, 2026). In this nationwide Welsh study, females diagnosed with ADHD in adolescence and early adulthood (aged 12–25 years) had significantly higher rates of hospital admissions and emergency department (ED) visits than those diagnosed in childhood (aged 5–11 years) (Martin et al., 2024, 2026). Hospitalisations were often related to mental health crises, injuries, and self‐harm. Clinically, those presenting with crisis or self‐harm often have other comorbidities that seem unrelated to ADHD. However, delayed diagnosis may result in greater dependence on acute support (Martin et al., 2026). Later diagnosis was also associated with higher rates of psychotropic medication prescriptions, indicating complex psychiatric presentations and greater support needs (Martin et al., 2026). That study's HSU variables included general practitioner, outpatient, inpatient, and ED contacts. No differentiation was made for those with or without intellectual disability (ID), despite high rates of co‐occurrence with ADHD and higher HSU compared to those without (Kreps et al., 2026; Perera et al., 2022; Sheehan et al., 2024).

Given the empirical‐evidence gap investigating the mental health burden for females with early versus late ADHD diagnosis, this study harnessed national quantitative data to investigate the implications of ADHD diagnostic timing on a range of mental and general HSU interactions. A national cohort of young adult females aged 18–24 years was established and investigated in both unadjusted and adjusted regression analyses, accounting for key confounding variables. Additionally, recognising the changing association between ID and HSU over age, analyses were stratified by ID.

## METHODS

### Study design and participants

This was a population‐based cohort study, utilising routinely collected de‐identified and individual‐level linked administrative data contained within NZ's Integrated Data Infrastructure (IDI). Females aged 18–24 years between July 1, 2021, to June 30, 2022, were included, based on the IDI estimated resident population (Gibb et al., 2016). This narrow age range minimised age‐related confounding associated with HSU and was restricted to the World Health Organization's upper limit for youth.

### Primary measures

*ADHD‐index* used previously established and employed case methods for IDI‐based data (Bowden et al., 2026; Bowden, Gibb, et al., 2020; Mujoo et al., 2023). Case identification was made by drawing on diagnostic codes alongside pharmaceutical dispensing information obtained from four health datasets. Specifically, the International Classification of Disease 10th revision Australian modification (ICD‐10‐AM) codes from inpatient hospitalisation data in the National Minimum Dataset (NMDS); Diagnostic and Statistical Manual of Mental Disorders, 4th edition (DSM‐IV) and ICD‐10‐AM codes from specialist mental health service use data in the Program for the Integration of Mental Health Data (PRIMHD); diagnosis codes from the point of referral to disability support services held in Socrates; and, medication inference from community pharmacy dispensing within the Pharmaceutical Collection (Pharms) data. ADHD‐index was identified within any of these four datasets, and timing was set to the earliest index date within or between datasets. ADHD‐index age (in years) was determined by subtracting participants birth date from this index date, and age groupings were informed by Siddiqui et al. (2024) employed thresholds (which were: 0–11, 12–17, and 18+ years) together with the empirical distribution observed here.

*ID‐index* was also based on previously established case methods for IDI‐based data (Bowden, Thabrew, et al., 2020). Diagnostic information was sourced from NMDS, Socrates, and PRIMHD. ID‐index was determined by presence of ID diagnostic codes across any of those datasets.

Six sentinel HSU variables were investigated, namely: *psychotropic pharmaceutical dispensing*; *psychiatric inpatient stays*; *psychiatric outpatient visits*; *admissions for self‐harm*; *non‐psychiatric hospitalisations*; and, *potentially avoidable hospitalisations*. These variables have been defined and employed elsewhere (McLay et al., 2025). All HSUs between the two‐year period July 1, 2022, to June 30, 2024, were categorised as binary indicators (0 vs. 1+ interactions). For HSU variables with multiple interactions, the HSU interaction date was assigned to the earliest recorded interaction for that variable within the two‐year period.

Pharms indicated pharmaceutical dispensing and differentiated between psychotropic and non‐psychotropic medications (McLay et al., 2022, 2025). Program for the Integration of Mental Health Data indicated psychiatric inpatient stays and psychiatric outpatient visits. NMDS established indicators for self‐harm admissions,non‐psychiatric hospitalisations, and potentially avoidable hospitalisations. Self‐harm admissions were defined by an ICD‐10‐AM codes for intentional self‐harm (X60‐X84 or Y870)—including self‐inflicted poisoning and injuries. Non‐psychiatric hospitalisations included all inpatient stays contained within NMDS but excluded mental health‐related events based on health specialty coding. The methodology for deriving potentially avoidable hospitalisations for youth in NZ was developed by the Ministry of Health (2020), and based on indicators for 16 main categories (e.g., respiratory conditions), each with subcategories and specific ICD‐10‐AM codes.

### Sociodemographic variables

Five sociodemographic variables were included: *sex*, *age*, and *ethnicity*—obtained from the IDI personal details table, together with *NZ Index of Deprivation*, *2018* (NZDep), and *location*—derived from residence information in the address notification table, measured at June 30, 2022. Due to the current unavailability within the administrative data, gender identity could not be examined. Thus, biological sex was used and categorised as male or female, with females eligible. Age (in years) was determined as at June 30, 2022, and calculated from birth date. Aligning with NZ's preferred method, total ethnic identification was employed whereby participants could identify with one or more groups. Level 1 Statistics New Zealand (SNZ) classifications were used, including: Māori (NZ's indigenous people), Pacific, Asian, European (including NZ Pākehā), Middle Eastern/Latin American/African, and Other (the latter two categorises combined). NZDep is an area‐level deprivation measure based on areas of approximately 60–200 residents, consolidating census information on various domains, such as employment, income, education level and home ownership into a single continuous index (Atkinson et al., 2019). As commonly practiced, this index was partitioned into quintiles, with one denoting the least deprived and five signifying the highest deprivation level. Finally, location was based on SNZ's urban/rural classification, with rural areas representing geographical locations populations typically <1000 individuals.

### Procedure

The IDI compiles administrative records of individuals' interactions with various government agencies across domains such as health, social services, education, justice, geography and housing. It also incorporates data from the 2013, 2018, and 2023 Censuses. These data sources are linked using established methodologies, aiming to match entities across datasets with a high accuracy (Shlomo, 2019; Statistics New Zealand, 2014). The process prioritises a high linkage rate while maintaining a false positive rate <2%, made possible by the detailed nature of selected variables (Kvalsvig et al., 2019). Core linking variables include first and last name, sex, and full birth date, supplemented by additional identifiers where available. Access to and use of the IDI data is governed by SNZ's ‘Five Safes’ framework for statistical disclosure control (Statistics New Zealand, 2024). Data access for this study was provided by SNZ under the security and confidentiality provisions of the Data and Statistics Act 2022. Only approved bona fide researchers undertaking projects considered to be in the public's interest within a secure accredited data laboratory can access this data. All results released from these secure laboratories undergo confidentiality checks by SNZ. The study proposal and protocols were approved by SNZ (MAA2017‐16).

### Statistical analysis

Reporting was guided by The Reporting of studies Conducted using Observational Routinely collected health Data statement (Benchimol et al., 2015). Initially, participant numbers and sociodemographic characteristics were described; followed by descriptions of the ADHD‐index and ID‐index variables. This comprised a histogram of ADHD index frequency across age, together with a corresponding Kaplan‐Meier curve that accounts for variation in the ADHD‐index over the participant age range. ADHD‐index age groupings were formed and compared to the sociodemographic variables using Fisher's exact test. An age effect between ID‐index and ADHD‐index was also assessed using Fisher's exact test. Next, the HSU variables were described, and pairwise correlations (*r*) estimated. Strength of *r* was interpreted using Cohen's guidelines, with 0.10 ≤ *r* < 0.30 considered small, 0.30 ≤ *r* < 0.50 considered medium, and 0.50 ≤ *r* considered large (Cohen, 1988). Consistent with the SNZ's statistical disclosure control policy (Statistics New Zealand, 2024), variables having any value level with a count <6 were suppressed (although remaining within the statistical analysis). Unadjusted and adjusted regressions of those with ADHD‐index followed, treating those with early aged ADHD‐index as the reference group. Because HSU was common, modified Poisson regression models (with log‐link function and robust variance estimators) were employed, and incident rate ratios (IRRs) together with 95% confidence intervals (CIs) were reported (Zou, 2004). Due to the strong ID‐index effect which changed over ADHD‐index age groups, all regression analyses included ID‐index and ID‐index × ADHD‐index group interaction terms, regardless of statistical significance. The adjusted model additionally included the measured sociodemographic terms. As complete case numbers were high, sensitivity analyses with imputed missing data were not conducted. However, two sensitivity analyses were conducted excluding psychiatric HSU contacts within ± 30 and ± 90 days of the ADHD‐index date to minimise misclassification of assessment‐related contacts as outcomes. All analyses were performed using Stata MP version 16.1 (StataCorp LLC, College Station, TX, USA), and two‐tailed *α* = 0.05 defined significance.

## RESULTS

### Participants

The IDI dataset contained 212,385 females aged 18–24 years within the eligible study period. There were no exclusions.

### Sociodemographic characteristics

The mean age of the sample was 21.5 years (range 18.0–24.9 years). Overall, almost one‐quarter (*n* = 52,122; 24.5%) of females identified as Māori, a relatively greater proportion resided in the most deprived areas (*n* = 56,562; 26.6%), and most were urban dwelling (*n* = 186,486; 87.8%). Table 1 provides the sociodemographic characteristics of the overall sample.

**TABLE 1:** Sociodemographic characteristics of the sample partitioned by ADHD‐index classification.

		ADHD‐index^a^			
	Non‐ADHD	Earlier	Mid	Later	Total
	*n* (%)	*n* (%)	*n* (%)	*n* (%)	*n* (%)
Ethnicity^b^					
Māori	51,093 (24.7)	411 (25.1)	333 (21.1)	285 (14.0)	52,122 (24.5)
Pacific	28,830 (13.9)	72 (4.4)	57 (3.6)	57 (2.8)	29,016 (13.7)
Asian	32,193 (15.5)	42 (2.6)	72 (4.6)	216 (10.6)	32,523 (15.3)
European	135,390 (65.4)	1446 (88.3)	1398 (88.8)	1779 (87.6)	140,013 (65.9)
Other	4935 (2.4)	24 (1.5)	36 (2.3)	75 (3.7)	5070 (2.4)
NZDep^c^					
1 (least dep.)	31,578 (15.3)	252 (15.4)	300 (19.2)	438 (21.6)	32,568 (15.4)
2	35,505 (17.3)	279 (17.1)	312 (20.0)	459 (22.7)	36,555 (17.3)
3	38,511 (18.7)	330 (20.2)	327 (20.9)	429 (21.2)	39,597 (18.7)
4	45,087 (21.9)	360 (22.0)	324 (20.7)	399 (19.7)	46,170 (21.8)
5 (most dep.)	55,548 (26.9)	414 (25.3)	300 (19.2)	300 (14.8)	56,562 (26.7)
Location^d^					
Urban	181,788 (88.1)	1431 (87.4)	1389 (88.7)	1878 (92.7)	186,486 (88.2)
Rural	24,489 (11.9)	207 (12.6)	177 (11.3)	147 (7.3)	25,020 (11.8)
Intellectual disability (ID)					
No	205,614 (99.3)	1275 (77.8)	1473 (93.5)	2013 (99.1)	210,375 (99.1)
Yes	1527 (0.7)	363 (22.2)	102 (6.5)	18 (0.9)	2010 (0.9)

Abbreviation: ADHD, attention‐deficit/hyperactivity disorder.

^a^ Earlier defined as having ADHD‐index at age < 12 years; Mid at age 12–17 years; and Later at age 18–24 years.

^b^ Females can identify with one or more ethnicities, so the total percentage sums greater than 100%.

^c^ Observations missing for 912 (0.4%) females.

^d^ Observations missing for 864 (0.4%) females.

### Attention‐deficit hyperactivity disorder (ADHD)

**FIGURE 1:**
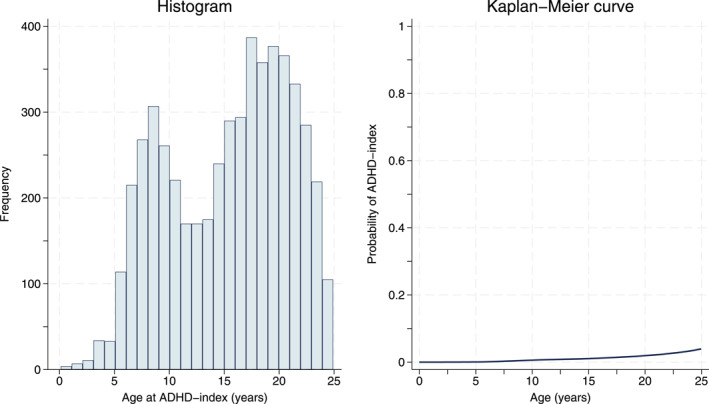
Histogram of ADHD‐index frequency over age (LHS) together with the associated Kaplan‐Meier probability of ADHD‐index (RHS) for the 212,385 females aged 18–24 years. ADHD, attention‐deficit/hyperactivity disorder.

In the analytical sample, 5244 (2.5%) females had an ADHD‐index, while 207,141 (97.5%) females had no indication (termed ‘non‐ADHD’). Figure 1 presents the histogram of ADHD‐index frequency over age. A bimodal pattern emerged, with indexing peaking at approximately age 8–10 years, and then again at approximately age 18–21 years. The apparent decline in ADHD‐index after age 21 years likely reflects the right‐censored nature of the cohort; that is, the effect of some younger participants classified as non‐ADHD, who would have been indexed by age 25 years. The gradient of the Kaplan‐Meier curve (Figure 1) over the age 21–24 years range does not suggest such a decline.

Based on this bimodal histogram, ADHD‐index was categorised into three age groupings, namely: age < 12 years (*n* = 1638; termed ‘Earlier’), age 12–17 years (*n* = 1575; termed ‘Mid’), and age 18–24 years (*n* = 2031; termed ‘Later’). Table 1 also presents the sociodemographic characteristics of the sample partitioned by these ADHD‐index groupings. Significant patterns emerged. Compared to the Later ADHD‐index group, the proportion of Māori females in the Earlier ADHD‐index group was higher (25.1% vs. 14.0%, *p* < .001), as was those from the most deprived areas (25.3% vs. 14.8%, *p* < 0.001). Conversely, the proportion of Asian females in the Earlier ADHD‐index group was lower than those in the Later ADHD‐index group (2.6% vs. 10.6%, *p* < .001), as was those from the least deprived areas (15.4% vs. 21.6%, *p* < .001).

### Intellectual disability (ID)

The sample included 2010 (0.9%) females with an ID‐index, of whom 486 (24.2%) were also ADHD‐indexed. There was a strong age association between females with dual ID‐index and ADHD‐index (*p* < .001), with 22.2% of those with ADHD‐index aged < 12 years also having ID‐index, decreasing to 0.9% for those aged 18–24 years; see Table 1. Given this strong age relationship between ID‐index and ADHD‐index, and the primary research question of interest, reporting of pursuant analyses were partitioned by ID‐index.

### Health service utilisation and clinical outcomes

Table 2 provides the observed frequencies (%) of non‐ADHD and ADHD‐index groups against the considered HSU outcome measurements, stratified by ID‐index. Overall, for the unsuppressed numbers, 56,226 (26.5%) females had psychotropic pharmaceutical dispensing, 48,900 (23.0%) were admitted to hospital for non‐psychiatric treatment, 15,333 (7.2%) experienced potentially avoidable hospitalisations, 14,169 (6.7%) had psychiatric outpatient visits, 1929 (0.9%) had psychiatric inpatient stays, and 1860 (0.9%) had hospital admissions for self‐harm. Apart from the relationship between potentially avoidable hospitalisations and non‐psychiatric hospitalisations (*r* = 0.50 for those without ID‐index and *r* = 0.63 with ID‐index), all other pair‐wise correlations between the considered outcome variables were *r* < 0.50. Outcomes frequencies (%) appeared to vary by ADHD‐index groups and by the presence of ID‐index; see Table 2.

**TABLE 2:** Observed frequencies (%) of non‐ADHD and ADHD‐index groups against the considered outcome measurements, stratified by ID‐index status.

	Without ID‐index				With ID‐index			
	Non‐ADHD	Earlier ADHD‐index	Mid ADHD‐index	Later ADHD‐index	Non‐ADHD	Earlier ADHD‐index	Mid ADHD‐index	Later ADHD‐index
	*n* (%)	*n* (%)	*n* (%)	*n* (%)	*n* (%)	*n* (%)	*n* (%)	*n* (%)
Psychotropic pharmaceutical dispensing								
No	153,030 (74.4)	609 (47.8)	549 (37.3)	717 (35.6)	1020 (66.8)	156 (43.0)	57 (55.9)	S
Yes	52,584 (25.6)	666 (52.2)	921 (62.7)	1296 (64.4)	507 (33.2)	207 (57.0)	45 (44.1)	S
Psychiatric inpatient stay								
No	203,904 (99.2)	1242 (97.4)	1410 (95.9)	1965 (97.6)	1482 (97.1)	339 (93.4)	96 (91.4)	S
Yes	1710 (0.8)	33 (2.6)	60 (4.1)	48 (2.4)	45 (2.9)	24 (6.6)	9 (8.6)	S
Psychiatric outpatient visit								
No	192,852 (93.8)	996 (77.9)	1092 (74.1)	1653 (82.0)	1287 (84.3)	261 (72.5)	69 (67.6)	9 (50.0)
Yes	12,762 (6.2)	282 (22.1)	381 (25.9)	363 (18.0)	240 (15.7)	99 (27.5)	33 (32.4)	9 (50.0)
Admission for self‐harm								
No	203,928 (99.2)	1245 (97.9)	1416 (96.1)	1977 (98.2)	1500 (98.2)	348 (95.1)	93 (91.2)	S
Yes	1686 (0.8)	27 (2.1)	57 (3.9)	36 (1.8)	27 (1.8)	18 (4.9)	9 (8.8)	S
Non‐psychiatric hospitalisation								
No	158,832 (77.2)	834 (65.4)	1008 (68.4)	1530 (76.0)	1008 (65.9)	213 (58.7)	57 (54.3)	9 (50.0)
Yes	46,782 (22.8)	441 (34.6)	465 (31.6)	483 (24.0)	522 (34.1)	150 (41.3)	48 (45.7)	9 (50.0)
Potentially avoidable hospitalisation								
No	191,220 (93.0)	1089 (85.4)	1284 (87.2)	1836 (91.2)	1245 (81.4)	288 (79.3)	81 (77.1)	S
Yes	14,397 (7.0)	186 (14.6)	189 (12.8)	177 (8.8)	285 (18.6)	75 (20.7)	24 (22.9)	S

*Note*: S denotes suppressed cells.

Abbreviation: ADHD, attention‐deficit/hyperactivity disorder.

Unadjusted regression analyses followed, limiting the sample to those who had an ADHD‐index (*n* = 5244), and treating females without ID‐index and having an Earlier ADHD‐index as the reference group. Table 3 presents the unadjusted modified Poisson regression IRR estimates, together with associated 95% CIs, of ADHD‐index groups against the considered outcome measurements. A significant ADHD‐index group association was found across all regressions (all *p* < .001). Similarly, the ID‐index × ADHD‐index group interaction was significant in all regressions (all *p* < .001 except non‐psychiatric hospitalisation *p* = .05), apart from the model of self‐harm admissions (*p* = .33) and potentially avoidable hospitalisations (*p* = .32). For participants without ID‐index, females in the Mid ADHD‐index group had significantly higher rates of psychotropic pharmaceutical dispensing, psychiatric inpatient stays, psychiatric outpatient visits, and self‐harm admissions compared to those in the Earlier ADHD‐index group. Apart from psychotropic pharmaceutical dispensing, estimated effect sizes for the Later ADHD‐index group were all dampened compared to the Mid ADHD‐index group. Compared to those in the Earlier ADHD‐index group, participants in the Later ADHD‐index group had significantly lower levels of psychiatric outpatient visits, non‐psychiatric hospitalisations and potentially avoidable hospitalisations.

**TABLE 3:** Unadjusted and adjusted modified Poisson regression incidence rate ratio (IRR) estimates, together with associated 95% confidence intervals (CIs), of ADHD‐index groups against the considered outcome measurements, stratified by ID‐index status.

	Without ID‐index			With ID‐index		
	Earlier ADHD‐index	Mid ADHD‐index	Later ADHD‐index	Earlier ADHD‐index	Mid ADHD‐index	Later ADHD‐index
	IRR (95% CI)	IRR (95% CI)	IRR (95% CI)	IRR (95% CI)	IRR (95% CI)	IRR (95% CI)
Psychotropic pharmaceutical dispensing						
Unadjusted	1 (reference)	1.20 (1.12, 1.28)	1.23 (1.16, 1.31)	1.09 (0.98, 1.21)	0.85 (0.69, 1.07)	1.60 (1.29, 1.97)
Adjusted	1 (reference)	1.17 (1.06, 1.29)	1.18 (1.04, 1.35)	1.11 (1.00, 1.23)	0.88 (0.70, 1.10)	1.52 (1.19, 1.95)
Psychiatric inpatient stay						
Unadjusted	1 (reference)	1.55 (1.03, 2.35)	0.89 (0.58, 1.38)	2.38 (1.42, 3.98)	2.55 (1.16, 5.61)	10.4 (4.61, 23.5)
Adjusted	1 (reference)	2.07 (1.20, 3.58)	1.48 (0.71, 3.09)	2.19 (1.30, 3.71)	2.92 (1.29, 6.62)	12.9 (4.69, 35.7)
Psychiatric outpatient visit						
Unadjusted	1 (reference)	1.17 (1.02, 1.34)	0.81 (0.71, 0.94)	1.26 (1.04, 1.53)	1.41 (1.04, 1.91)	2.52 (1.65, 3.86)
Adjusted	1 (reference)	1.08 (0.88, 1.32)	0.75 (0.57, 0.98)	1.21 (1.00, 1.48)	1.23 (0.89, 1.70)	1.94 (1.22, 3.07)
Admission for self‐harm						
Unadjusted	1 (reference)	1.70 (1.10, 2.65)	0.79 (0.48, 1.28)	2.06 (1.14, 3.70)	3.84 (1.87, 7.90)	4.89 (1.26, 18.9)
Adjusted	1 (reference)	1.01 (0.55, 1.85)	0.38 (0.17, 0.83)	2.09 (1.14, 3.83)	2.40 (1.12, 5.14)	2.05 (0.49, 8.53)
Non‐psychiatric hospitalisation						
Unadjusted	1 (reference)	0.91 (0.82, 1.02)	0.70 (0.63, 0.78)	1.20 (1.04, 1.38)	1.32 (1.06, 1.65)	1.45 (0.91, 2.31)
Adjusted	1 (reference)	1.20 (1.02, 1.41)	1.09 (0.87, 1.35)	1.11 (0.96, 1.29)	1.52 (1.20, 1.92)	1.87 (1.12, 3.12)
Potentially avoidable hospitalisation						
Unadjusted	1 (reference)	0.87 (0.72, 1.05)	0.60 (0.50, 0.73)	1.40 (1.10, 1.78)	1.53 (1.04, 2.25)	1.52 (0.64, 3.65)
Adjusted	1 (reference)	0.75 (0.57, 1.00)	0.49 (0.34, 0.71)	1.37 (1.07, 1.75)	1.29 (0.86, 1.93)	1.07 (0.43, 2.64)

*Note*: adjusted regression models included: age (years), ethnicity, NZDep, location, ID‐index, and ID‐index × ADHD‐index group interaction.

Abbreviations: ADHD, attention‐deficit/hyperactivity disorder; CIs, confidence intervals; IRR, incidence rate ratio.

Females with ID‐index and in the Earlier ADHD‐index group had higher estimated IRRs across all measured variables, compared to their counterparts without ID‐index; and, significantly so for psychiatric inpatient stays, and psychiatric outpatient visits, self‐harm admissions, non‐psychiatric hospitalisations, and potentially avoidable hospitalisations. Unlike the pattern observed for females without ID‐index, those with ID‐index and in the Later ADHD‐index group had higher estimated IRRs compared to those in the Earlier or Mid ADHD‐index groups across all considered HSU variables; see Table 3.

### Adjusted regression analyses

Complete case (*n* = 5,226, 99.7%) modified Poisson regression analyses were then conducted, adjusting for age (years), ethnicity, NZDep, location, ID‐index, and the ID‐index × ADHD‐index group interaction. In addition to the unadjusted results, Table 3 includes the adjusted IRRs and associated 95% CIs. A significant ADHD‐index group association was found across all adjusted regressions (all *p* < .001, except for psychotropic pharmaceutical dispensing *p* = .01, and non‐psychiatric hospitalisations *p* = .02). Similarly, the ID‐index × ADHD‐index group interaction was significant in all adjusted regressions (all *p* < .01), except for the models of self‐harm admission (*p* = .41), non‐psychiatric hospitalisations (*p* = .18), and potentially avoidable hospitalisations (*p* = .42). While some differences in the unadjusted and adjusted IRR effect size estimates can be observed in Table 3, the overall patterns remained similar. The most notable change was for self‐harm admission between Mid and Earlier groups for those without ID between the significant unadjusted (IRR = 1.70; 95% CI: 1.10, 2.65) and the non‐significant adjusted (IRR = 1.01; 95% CI: 0.55, 1.85) estimates.

### Sensitivity analyses

Adjusted modified Poisson regression IRR, together with associated 95% CIs, for the complete case analysis (*n* = 5244), and in the sensitivity analyses excluding those with ID‐index indication and first psychiatric HSU outcome date being ≤ 30 days apart (*n* = 5196; 48 females excluded) or ≤ 90 days apart (*n* = 5109; 135 females excluded) were conducted and graphically presented in Supporting Information S1: Figures S1–S6 within Appendix S1 of the supporting information. Each of these figures revealed stable IRR estimates between scenarios and a high degree of 95% CI overlap.

## DISCUSSION

In this large, national NZ study several notable findings emerged. Overall, 2.5% of females were ADHD‐indexed, well short of the 6%–10% of children and adolescents with ADHD worldwide (Ayano et al., 2023). This underscores the gender‐difference in diagnosis reported elsewhere (Almekhlafi & Jain, 2024; Attoe & Climie, 2023; Martin et al., 2024). Moreover, age of ADHD‐index was bimodal, suggesting separate diagnostic drivers. The first peak, between 8 and 10 years, likely reflects primary school identification initiated by caregivers and teachers (Staff et al., 2023), coupled with the clinical requirements and time‐course needed for diagnosis (Ministry of Health, 2025b). The second peak, between 18 and 21 years, may result from self‐initiated diagnosis after a period of undiagnosed ADHD or masking (Martin, 2024; Morgan, 2023; Young et al., 2020). Overall, 2031 (38.7%) females with ADHD‐index were indexed at ages 18–24 years, outside the typical diagnosis age range given in the Ministry of Health's June 2025 Clinical Principles Framework (Ministry of Health, 2025b).

The age‐dependent relationship between ADHD and ID was also notable with 22.2% of those ADHD‐indexed at aged <12 years also having ID‐index, decreasing to 0.9% for those aged 18–24 years. This implies females with both ADHD and ID appear to be diagnosed earlier than those with ADHD alone. Young children with ID often show behaviors such as inattention, impulsivity, and hyperactivity. Children exhibiting these behaviors are more likely referred for evaluation, increasing their likelihood of early ADHD diagnosis (Tam et al., 2025; Young et al., 2020). Conversely, diagnostic overshadowing (e.g., behaviors attributed to cognitive or functional issues as opposed to ADHD) may act to reduce the likelihood of ADHD diagnosis during adolescence.

In terms of mental HSU among those without ID‐index, rates of psychotropic pharmaceutical dispensing, psychiatric inpatient stays, and non‐psychiatric hospitalisations were significantly higher in those with ADHD‐index at age 12–17 years compared to those <12 years in adjusted analyses. Indeed, the estimated IRR for psychiatric inpatient for these females was over double. While ADHD‐index age group membership likely reflects multiple confounding factors, counterfactual evidence on the impact of earlier diagnosis in those aged 12–17 years is unavailable. Nonetheless, if the excess HSU observed between age groups is attributable to delayed diagnosis, the associated individual burden and societal costs appear substantial. Effect size estimates for psychiatric outpatient visits and self‐harm admissions were also significantly higher among those ADHD‐indexed at age 12–17 years in the unadjusted analyses, but were both confounded within the adjusted analyses. Although these IRR estimates remained non‐significantly higher than those in the <12‐year group, effect sizes were small and unlikely to be clinically meaningful.

Compared to those ADHD‐indexed at age <12 years without ID‐index, females ADHD‐indexed at age 18–24 years without ID‐index also had significantly higher rates of psychotropic pharmaceutical dispensing, but significant lower rates of psychiatric outpatient visits, self‐harm admissions, and potentially avoidable hospitalisations. These females may be more likely to have co‐occurring mental health conditions, resulting in higher rates of psychotropic medication use (Attoe & Climie, 2023; Martin et al., 2026; Siddiqui et al., 2024). However, females diagnosed with ADHD in early adulthood may represent a subgroup with less severe neurodevelopmental burden and different service trajectories, which could partially explain lower subsequent psychiatric and hospital service use (Barclay et al., 2024; Martin et al., 2026). Further investigation is required to understand these associations, but they may result from a combination of symptom presentation, coping strategies, and healthcare engagement patterns. Some evidence suggests that late diagnosis ADHD is associated with relatively higher intelligence, contributing to better overall health outcomes and reduced hospitalisation risk (Hare et al., 2024; Wraw et al., 2015). Additionally, while ID was explicitly identified and controlled for here (and younger ADHD‐indexed groups found to have higher ID‐indexed co‐occurrence), it is likely that earlier ADHD‐indexed participants also have higher co‐occurrence rates of other unmeasured conditions, such as autism spectrum disorder, which are associated with higher HSU (McLay et al., 2025; Tam et al., 2025; Zablotsky et al., 2020).

Finally, when considering ID‐indexed females with ADHD‐index at age <12 years, their mental HSU rates were significantly higher than those without ID‐index at the same age. Dual ID and ADHD diagnoses elevates clinical complexity, increasing the demand for psychological support (Clark & Bélanger, 2018; Perera et al., 2022). Females with ID‐index and ADHD‐index at age 18–24 years recorded the highest mental HSU rates across all measures. Compared to females without ID‐index at age <12 years, those ID‐indexed at the same ADHD‐index age had psychiatric inpatient stays with rates over two times higher (IRR = 2.19; 95% CI: 1.30, 3.71), and for those ID‐indexed at age 18–24 years the rates were nearly 13 times higher (IRR = 12.9; 95% CI: 4.69, 35.7). This reflects the downstream impact of delayed ADHD diagnosis combined with co‐occurring ID, which worsens general functioning, and ultimately, heightening the need for care (Bastos Maia & Martins Miranda, 2025; Kreps et al., 2026; Martin et al., 2026; Perera et al., 2022; Siddiqui et al., 2024).

### Strengths and limitations

The study has several strengths, including the curation of a general population prospectively collected research dataset, linking an apposite suite of robustly defined primary and potentially confounding variables. Individual‐level data, matched with high linkage probabilities, were analyzed, mitigating the possible ecological fallacy bias. Careful attention was given to ID identification and pursuant statistical analyses and reporting. If ID‐indexed females were pooled with those without ID‐index, the impact of index age on HSU would have been inflated compared to the without ID‐index estimates reported here.

However, the study also had limitations. Inferring ADHD based on medication records can be problematic, as not everyone with ADHD is medicated, and ADHD medications may be prescribed for off‐label uses. While widely used (Bowden, Gibb, et al., 2020; Mujoo et al., 2023), the ADHD and ID case identification relied on an unvalidated methods which may have introduced misclassification through incomplete capture of cases or inclusion of non‐cases (Bowden et al., 2026). Further research establishing the psychometric properties of these identification algorithms would increase the robustness of such epidemiological studies. A small number of participants aged 18–24 years within the non‐ADHD group were likely to be misclassified, as they may have received an ADHD diagnosis after the study end date but before they turned 25 years. Although, this unlikely affected the analyses of ADHD‐indexed females, other than to reduce numbers and statistical power. Additionally, as noted earlier, ADHD‐index age group membership is likely dependent on characteristics connected to both diagnostic timing and HSU (e.g., ADHD presentation, severity, psychiatric co‐occurrences) which may limit direct group comparability (Tam et al., 2025). Thus, an important interpretative consideration is the heterogeneity of ADHD diagnoses across developmental stages. Diagnoses first made in young adulthood may not uniformly reflect delayed identification of a single neurodevelopmental phenotype, particularly among females, where later diagnosis often occurs in the context of longstanding internalising symptoms and complex clinical histories. Consequently, some adult diagnoses may reflect diagnostic practice and clinical context as much as developmental continuity. Framing ADHD as a heterogeneous construct helps temper causal inference and avoid diagnostic essentialism. Residual confounding may also introduce estimation bias (Fewell et al., 2007). Biological (e.g., physical illnesses), social (e.g., support networks), psychological (e.g., personality traits), and historical (e.g., health system interactions) factors were unavailable and unaccounted for within the statistical models. Moreover, comorbidity imbalance between diagnostic timing groups may introduce differential non‐systematic bias as later‐diagnosed females may enter services via anxiety, depression, or trauma pathways, which themselves drive higher HSU. Lastly, mental HSU was interpreted primarily as an indicator of burden and unmet need; however, alternative explanations could be considered. Health service utilisation may also reflect differences in help‐seeking behavior, service accessibility, diagnostic vigilance, or patterns of follow‐up following diagnosis, rather than severity alone. Accordingly, observed differences in HSU should not be attributed to diagnostic delay alone, but understood as arising from the interplay between clinical complexity, access, and surveillance effects.

### Implications of the findings

Several implications arise from these findings. As this study is associative, causality between the timing of ADHD diagnosis and mental HSU cannot be inferred, and replication in longitudinal studies is needed before causal interpretations can be considered. Notably, a bimodal distribution of ADHD‐index was observed, with peaks at approximately 8–10 years and 18–21 years; individuals indexed between these peaks (aged 12–17 years) demonstrated higher rates of HSU across multiple measures. Compared to their earlier ADHD‐indexed counterparts, females aged 12–17 years at diagnosis were associated with notably higher rates of psychiatric inpatient stays (107% higher), non‐psychiatric hospitalisation visits (20% higher), and psychotropic pharmaceutical dispensing (17% higher). Arguably, on a population level, these are both statistically and clinically important. If these findings are replicated, it suggests that there may be females with ADHD in NZ who have been under‐recognised or overlooked at the first peak (Barclay et al., 2024; Martin et al., 2026; Morgan, 2023; Young et al., 2020). This was likely exacerbated by NZ's clinical workforce shortage which has hampered timely diagnosis and treatment (Ministry of Health, 2024). In an effort to ease workforce pressures and improve timely access to care, from February 2026 the Ministry of Health has extended prescribing and diagnostic authority so that GPs and nurse practitioners can initiate treatment for adults with ADHD, while nurse practitioners working within child health or mental health services can diagnose ADHD and commence treatment for children and adolescents (Ministry of Health, 2025a). The manner that this is operationalised and underpinned by professional development remains uncertain, as do the potential implications. Finally, while the use of NZ's IDI is a key strength here, the organisation of mental health services, diagnostic pathways, and prescribing practices may differ across countries, and this should be considered when assessing the generalisability of these findings to other health‐care contexts.

## CONCLUSION

Prioritising timely ADHD diagnosis in females may offer important individual and societal benefits. The findings from this national study suggest that many females in NZ experience delayed diagnosis, and that diagnosis during adolescence is associated with higher rates of HSU. Beyond addressing workforce capacity and referral pathway constraints, improved nuanced understanding of the gender‐specific presentation of ADHD is essential. Strategies to support timely diagnosis in females—such as increasing professional awareness of symptom masking and the impact of co‐occurring mental health conditions—are well recognised. However, delayed and missed diagnoses persist. Emerging evidence from this study and elsewhere suggests that more timely ADHD diagnosis may help mitigate both personal burden and broader societal costs. Collectively, these findings underscore the need for sex‐informed diagnostic approaches and service models that better support early ADHD identification in females.

## AUTHOR CONTRIBUTIONS

**Estelle Alderson:** Conceptualization; study design, interpretation of data; writing—original draft preparation; writing—review and editing. **Nicholas Bowden:** Conceptualization; study design; data curation; interpretation of data; writing—review and editing. **Laurie McLay:** Conceptualization; study design; interpretation of data; writing—review and editing. **Ben Beaglehole:** Writing—review and editing. **Philip J. Schluter:** Conceptualization; study design; data analysis; interpretation of data; writing—original draft preparation; writing—review and editing. All authors approved the final manuscript for publication.

## CONFLICT OF INTEREST STATEMENT

The authors declare no conflicts of interest.

## ETHICS CONSIDERATIONS

This study is a secondary analysis of routinely‐collected de‐identified administrative information housed and curated by SNZ. The IDI is designed to be a research database that holds de‐identified administrative records about people and households that come from government agencies, SNZ surveys, and non‐government organisations. Therefore, informed participant consent is not applicable. Based on NZ's Health and Disability Ethics Committees' (HDEC) checklist, the study did not meet the threshold required for formal ethics review. The University of Otago's Human Research Ethics Committee defined and approved the study as ‘Minimal Risk Health Research—Audit and Audit related studies’ (reference: HD17/004; date approved: 27 February 2017). All methods and results were performed in accordance with SNZ and HDEC guidelines and regulations.

## Supporting information

Supporting Information S1

## Data Availability

The datasets used for statistical analysis are held within the IDI and managed by SNZ. Data supporting this study's findings are available from SNZ, but restrictions apply to the availability of these data which were used under licence for the current study and are not publicly available. However, the data and code used are available from the authors upon reasonable request and with permission of SNZ (see: https://www.stats.govt.nz/integrated‐data/apply‐to‐use‐microdata‐for‐research/). These data can only be accessed by approved bona fide researchers, for approved projects that are in the public interest and within a secure accredited data laboratory.
