# Supplementary material for: Implications of attention‐deficit/hyperactivity disorder diagnostic timing on mental health service utilisation in young adult females: A population‐based record linkage cohort study
Source: JCPP Adv. 2026 Jun 3:e70133. Online ahead of print. doi: 10.1002/jcv2.70133 (PMC13339066; doi:10.1002/jcv2.70133)
Supplement: Supplementary file 1 — Supporting Information S1 [file JCV2-9999-e70133-s001.docx]

**Implications of attention-deficit/hyperactivity disorder diagnostic timing on mental health service utilisation in young adult females: a population-based record linkage cohort study**

**Supporting Information**

**Appendix S1. Sensitivity analyses**

Adjusted modified Poisson regression incidence rate ratio (IRR) estimates (box), together with associated 95% confidence intervals (lines) for the complete case analysis (n=5,244), and in the sensitivity analyses excluding those with ID-index indication and first outcome date being ≤30 days apart (n=5,196; 48 females excluded) or ≤90 days apart (n=5,109; 135 females excluded) were conducted and graphically presented in Figures S1-S6 below. Note, in each of these figures “Ref.” denotes the reference group (Earlier without ID) and IRR is given on a log scale.

**Figure S1** adjusted modified Poisson regression incidence rate ratio (IRR) estimates (box), together with associated 95% confidence intervals (lines), of psychotropic pharmaceutical dispensing for the complete case analysis (indicted by blue), and in the sensitivity analyses excluding those with ID-index indication first outcome date being ≤30 days apart (solid red line) or ≤90 days apart (dashed red line).

**Figure S2** adjusted modified Poisson regression incidence rate ratio (IRR) estimates (box), together with associated 95% confidence intervals (lines), of psychiatric inpatient stay for the complete case analysis (indicted by blue), and in the sensitivity analyses excluding those with ID-index indication first outcome date being ≤30 days apart (solid red line) or ≤90 days apart (dashed red line).

**Figure S3** adjusted modified Poisson regression incidence rate ratio (IRR) estimates (box), together with associated 95% confidence intervals (lines), of psychiatric outpatient visit for the complete case analysis (indicted by blue), and in the sensitivity analyses excluding those with ID-index indication first outcome date being ≤30 days apart (solid red line) or ≤90 days apart (dashed red line).

**Figure S4** adjusted modified Poisson regression incidence rate ratio (IRR) estimates (box), together with associated 95% confidence intervals (lines), of admission for self-harm for the complete case analysis (indicted by blue), and in the sensitivity analyses excluding those with ID-index indication first outcome date being ≤30 days apart (solid red line) or ≤90 days apart (dashed red line).

**Figure S5** adjusted modified Poisson regression incidence rate ratio (IRR) estimates (box), together with associated 95% confidence intervals (lines), of non-psychiatric hospitalisation for the complete case analysis (indicted by blue), and in the sensitivity analyses excluding those with ID-index indication first outcome date being ≤30 days apart (solid red line) or ≤90 days apart (dashed red line).

**Figure S6** adjusted modified Poisson regression incidence rate ratio (IRR) estimates (box), together with associated 95% confidence intervals (lines), of potentially avoidable hospitalisation for the complete case analysis (indicted by blue), and in the sensitivity analyses excluding those with ID-index indication first outcome date being ≤30 days apart (solid red line) or ≤90 days apart (dashed red line).
